# Navigating transitions: a qualitative study of nursing teams’ experiences of educational and cultural transitions in Germany

**DOI:** 10.1186/s12912-024-02383-0

**Published:** 2024-10-08

**Authors:** Marwa Schumann, Lisa Peppler, Patricia Beck, Liane Schenk

**Affiliations:** 1 Dieter Scheffner Center for Medical Education und Educational Research Dean´s Office for Study Affairs, Charité – Universitätsmedizin Berlin, Charitéplatz 1, 10117 Berlin, Germany; 2 Medical Education Department, Faculty of Medicine, Alexandria University, Alexandria, Egypt; 3 Institute of Medical Sociology and Rehabilitation Science, Charité - Universitätsmedizin Berlin, Berlin, Germany; 4 FOM University of Applied Sciences, IEGUS - Institute for European Health and Social Economy, Essen, Germany

**Keywords:** Nurse migration, Nursing teams, Academisation of nursing, Qualitative study

## Abstract

**Background:**

The global migration of health professionals in general and nurses in particular, has led to nursing shortages and socioeconomic impacts on health systems in both source and destination countries. Adding to the complexity of the situation is the fact that the nursing profession itself is evolving from a vocational to an academic one. Although nursing migration and academization have been studied from either an institutional or an individual perspective, there is a gap in the literature regarding how nursing teams experience these transitions. This study aims to explore how nursing teams navigate through the transitions of academization and internationalization of the current dynamic nursing landscape in Germany.

**Methods:**

Based on social constructivism epistemology this qualitative study involved face-to-face focus group discussions conducted at several hospital sites in Germany from September 2021 to May 2023. The focus group discussions were audio-recorded, transcribed, and analysed using content analysis; the four dimensions of transition theory according to Schlossberg’s Four S (4 S) framework (self, situation, support, and strategy) were used as a priori items to construct the coding framework.

**Results:**

Nine focus group discussions were conducted with a total of 40 nurses from different educational and migrant backgrounds. The analysis showed that the transition experiences of the nursing teams were heterogeneous, with educational and national backgrounds playing an important role in how realistic their expectations of their professional roles and identities were. The dynamic situation characterized by a shortage of qualified nursing staff, increases the pressure on nursing teams and underscores the importance of employer-provided and peer support. Onboarding and communication are key strategies used depending on the duration of the employee turnover.

**Conclusion:**

This study provides insights into the challenges and coping strategies of nursing teams in the current dynamic scene of migration, academicization and professional socialisation in Germany. Extending the Schlossberg 4 S framework from the individual to the team perspective provides a comprehensive view of the transitional experiences of nursing teams. Within each domain of the framework, the experiences of nursing teams are remarkably diverse. Educational background (vocational or academic) and origin (German or foreign trained) play an important role in shaping the transitional experiences of nursing teams.

**Supplementary Information:**

The online version contains supplementary material available at 10.1186/s12912-024-02383-0.

## Introduction

The global migration of health workers in general, and nurses in particular, has played a critical role in shaping the face of health care systems [1]. Over the past half century, the shortage of nurses has strained the health systems of several countries, with long-term political and socio-economic consequences [2]. One in eight nurses currently works in a country other than where they trained, and the World Health Organization (WHO) predicts that an additional 9 million nurses will be needed by 2030 to achieve the United Nations’ Sustainable Development Goal of universal health coverage [3–5].

Migration trends primarily follow economic disparities, with flows from less developed to more developed countries and from low-income to high-income countries [6]. The main destination countries, such as the United Kingdom and the United States, have consistently attracted nurses due to enduring factors at the macro level (national) and the meso level (professional). The main drivers include aspects such as remuneration, safety concerns, career opportunities, job satisfaction and factors related to the work environment [1]. In particular, these motivational factors appear to be consistent across nurses who have already migrated, those who express the intention to migrate, and across geographical regions within low- and middle-income countries (LMICs) [1]. Consequently, the political economy of regulating the conditions of entry for foreign nationals differs between traditional migrant-receiving countries (for example, the UK and the USA) and more recent migrant-receiving countries (for example, Germany and Austria), in which “legal, administrative and political mechanisms for controlling and regulating mass immigration” are still evolving [7]. This study aims to explore how nursing teams navigate through the transitions of academization and internationalization of the current dynamic nursing landscape in Germany as a newer migrant-receiving country.

In the following sections, we first provide an overview of the legal and policy frameworks regulating nurse mobility at the global, European and German levels, followed by an illustration of the different theories of transition, with a particular focus on Schlossberg’s 4 S theory, which is used as the framework for this study.

### Legal and policy frameworks

At the international policy level, several global agreements have been established to oversee the movement and recruitment of health workers. Examples include the World Health Organization’s (WHO) Global Code of Practice, the Health Worker Migration Policy Initiative (HWMPI) and the Global Health Workforce Alliance (GHWA), all launched in 2010 [8, 9]. Directive 2005/36/EC, later revised by Directive 2013/55/EU, sets out the rules for the recognition of professional qualifications in the EU Member States and outlines the specifications for the training of nurses, covering aspects such as curriculum content, work placements and time spent in clinical practice [10].

Like other European countries, Germany is facing a severe shortage of nurses; by 2030, Germany will need 520,000 full-time nursing positions which is particularly challenging given an ageing and increasingly chronically ill population, deteriorating working conditions in German hospitals and high burnout and job dissatisfaction among nurses [3, 11–13]. In 2019, almost as many internationally trained nurses came to Germany as were trained in Germany itself [14]. The countries of origin of international nurses in Germany range from Eastern, Southern and Western European countries to Russia, Southeast Asian countries, and South and Central American countries. The main countries of origin are Bosnia-Herzegovina (17.6%), Serbia (15.4%), the Philippines (13.6%), Albania (7.9%) and Romania (6.9%) [14].

Nursing migration to Germany is regulated by the Skilled Migration Act (Fachkräfteeinwanderungsgesetz), which came into force in 2020 and makes it easier for skilled workers, including nurses, from outside the European Union to immigrate to Germany to take up employment opportunities [15]. Guided by the WHO Global Code of Practice, European and German laws and regulations, the recruitment of foreign-trained nurses is carried out in two ways, either through the specialized department of the Federal Employment Agency or through private recruitment agencies [15]. In contrast to destination countries with a longer history of nurse migration, Germany began active recruitment only a decade ago. One example is the German government’s Triple Win project, which since 2013 has provided a framework for accompanied migration of nurses in the form of bilateral agreements [16].

In 2017, the enactment of the Nursing Care Reform Act marked a pivotal moment in the evolution of professional nursing education. Prior to this legislative development, the nursing profession had primarily been exclusively vocational. However, with the implementation of the Nursing Care Reform Act, a significant change occurred as the nursing profession expanded to include not only vocational but also academic components. With the establishment of the first comprehensive 3-year bachelor’s degree program, professional nursing education now comprises three main pathways: the 1-year nursing assistant vocational training program, the traditional 3-year nurse specialist vocational training program, and the newly established 3-year academic bachelor’s degree program. Therefore, the (partial) academization of nursing in Germany is only just beginning; only 1 to 2% of a German nursing cohort have completed an academic degree [17].

Although systematic or comparative statistics are rarely available, the academic background of nurses varies from country to country – from 88% in Thailand to 11% in the UK [18]. Therefore, most of the international nurses coming to Germany are academically trained. In theory, these nurses are more highly qualified than their colleagues with a national nursing qualification [19]. In practice, international migration always involves a reassessment of the qualifications and competences acquired in the country of origin. This reassessment is - especially in the health sector - depends on the current needs of the destination country [20]. The fact that the level of qualification of international and German nurses is nevertheless comparable [21] is the result of the practice of professional recognition in Germany, which for migrant nurses means a “paradoxical experience of recognition and devaluation at the same time” [22]. The process of recognition can take up to two years. During this time, the internationally trained nurses are only allowed to work as nursing assistants. However, even after full recognition, their new professional responsibilities and activities are usually very different from those they know from their country of origin.

Research on migrant nurses has mainly focused on the macro level, in particular on the drivers of migration and its impact on health systems [23–25]. Other studies have analyzed the experiences of migrant nurses and the institutions providing care at the micro or meso level, mostly using qualitative methods [16, 26]. Previous research has focused on the economic, epidemiological, and geographic aspects of nurse migration in Europe, but there is still a noticeable gap in research, particularly in the limited exploration of nursing teams, which are specifically understudied. In contrast to countries with a long history of nurse migration, Germany, which until recently had not considered migration as a possible solution for recruiting qualified nurses, has only lately experienced a sharp increase in foreign-trained nurses, making the evolving process worth exploring [27]. This study makes a novel contribution to the existing literature by shedding light on the integration of foreign-trained nurses into German nursing teams, an area that remains under-researched. While previous studies have predominantly focused on macro-level phenomena such as workforce shortages and policy implications, the present research explores the interpersonal and team dynamics within healthcare settings. By doing so, it offers valuable insights into the impact of nurse migration on team cohesion, communication, and overall job satisfaction, which are essential for ensuring effective patient care [13, 28]. Furthermore, this study identifies the obstacles encountered by internationally educated nurses in adapting to the German healthcare system, thus providing empirical evidence to inform policymakers and healthcare institutions on how to enhance integration practices.

Given the interconnectedness of European healthcare systems, exploring the German context could provide valuable insights that contribute to a broader European understanding of nursing team dynamics [11, 16, 29].

Therefore, this study aims to explore how nursing teams navigate through the transitions of academization and internationalization of the current dynamic nursing landscape in Germany.

### Theoretical background

The evolving transition of the nursing profession in Germany, influenced by internationalization and academization, presents significant challenges for nurses and nursing teams, making understanding and assessing adaptability to change crucial. There is an ongoing discourse in the literature and research about the optimal perspective from which to examine how individuals and teams deal with transitions, and several social science theories and frameworks have been developed to illuminate the experiences of both individual healthcare workers and teams. William Bridges’ Transition Model explores the psychological aspects of change and divides the process into stages of ending, neutral zone and new beginning [30]. Meleis’ transition theory, with a focus on nursing, examines how transitions affect health and well-being [31]. Altogether, these theories enrich our understanding of the multiple dimensions of transitions in healthcare settings, including emotional, psychological, and socio-cultural aspects for both individuals and teams.

Among these theories, Schlossberg’s 4 S framework is unique in that it provides a holistic perspective on change, taking into account a range of psychological, social and situational factors that can influence how one responds to change [32]. We chose this framework because it is particularly well suited to this study, providing a structured approach to exploring the experiences of nursing teams in response to the transitions of academization and internationalization. Traditionally applied to individual nurses, the dimensions of this framework-self, situation, support, and strategy-offer valuable insights when extended to the collective experiences of nursing teams [33]. The framework also allows for a comprehensive analysis of how individual characteristics, such as educational and national backgrounds, shape team dynamics and collective coping strategies within nursing teams. The emphasis on external factors allows for an understanding of how the broader nursing landscape, characterized by workforce shortages and evolving demands, affects team dynamics. In addition, the framework highlights the critical role of external supports, such as employer-provided training and peer support, and its focus on adaptive strategies offers insights into how teams adapt to factors such as turnover and length of stay, providing a deeper understanding of effective coping mechanisms in a dynamic healthcare environment. Several studies have used the 4 S framework to explore the transitions of individual nurses, such as the transition of international postgraduate nursing students enrolled in an MSc in Nursing program at a UK university [33], the transition of Enrolled Nurses (ENs) to Registered Nurses (RN) [34], the career transition of second-career students into professional nursing [35] and transitioning through a Bachelors nursing program [36].

On an individual level, the framework proposes that four distinct factors can significantly influence a person’s ability to effectively manage and cope with change: The ‘self-factor’, which is related to an individual’s personal characteristics, the ‘situational factor’, which is related to the inherent characteristics of the change itself; the ‘support factor’, which includes the availability of social support from others; and finally, the ‘strategies factor’, which involves the use of specific approaches designed to alleviate potential stressors that may arise during the management of change.

This study builds on the theory of Schlossberg’s 4 S framework but expands the concept to a supra-individual perspective by focusing on the transition processes of teams. The aim of this study is to explore how nursing teams navigate the dynamic interplay between migration and academization in a transitioning healthcare context in Germany, with the ultimate aim of providing a nuanced understanding of transitions in the nursing profession in Germany, considering the interplay of socio-cultural, educational and structural factors. Our research question is “How do nursing teams in Germany navigate the transitions of academization and internationalization in the current nursing landscape, and what factors within the team context (self, situation, support, and strategies) influence their experiences?”

## Methods

### Study design and setting

As part of the research project Care in Transition - Nursing Teams in the Area of Conflict between Migration and Academization (CareTrans), this study explores the impact of the changing educational, professional, and cultural landscape of the health care professions in Germany on the dynamics of nursing teams.

Underpinned by the epistemology of social constructivism that considers knowledge as being constructed by individuals through social interactions and cultural experiences, a qualitative approach was adopted to explore how nursing teams navigate through the transitions of academization and internationalization of the current dynamic nursing landscape in Germany as being ‘constructed through social interaction’ [37]. The focus group discussion is the most suitable survey method for reconstructing typical collective orientation patterns for a team [38].

Data collection took place in a natural setting, ensuring minimal disruption to participants’ daily routines by face-to-face focus group meetings, across multiple hospital sites in Germany from September 2021 to May 2023 [39].

Participants were recruited using a purposive, maximum variation sampling strategy to explore a wide range of experiences and maximize the chances of eliciting data [40, 41]. Recruitment was mainly done by e-mail or in person during the participant observation that took place before the group discussions (participant observation data is beyond the scope of our research question and will be included in upcoming articles).

Focus groups are either homogeneous or heterogeneous, depending on the socio-cultural, educational and professional background of the participants and their experiences and attitudes [41, 42]. In the current study, we used a mix of homogeneous and heterogeneous focus group constellations to enrich the discussions with a wide range of perspectives and to provide a rich, varied and comprehensive insight into the subject of the study. Homogeneous focus groups included either only migrant nurses (F, I) or only German nurses (G), while heterogeneous focus groups included both migrant and German nurses (H). (see Table 1). German nurses are those who were born in Germany and migrant nurses are those who were born abroad (or whose parents were born abroad) [43, 44]. While some focus groups included teams working in the same hospital but not on the same wards, others consisted of team members who worked together on a daily basis (A-E) (see Table 1).

### Qualitative data collection, transcription and translation

**Fig. 1:**
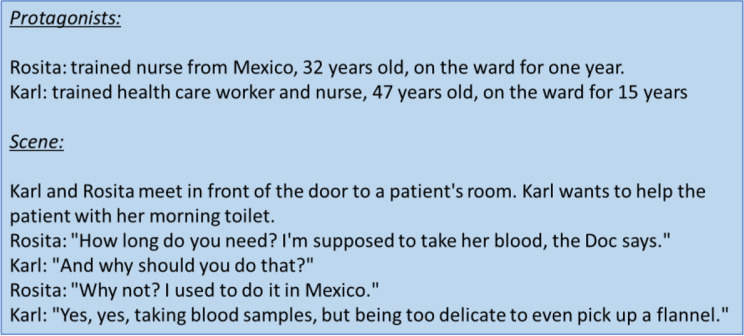
Discussion stimulus CareTrans: Rosita and Carl

Data collection and analysis took place simultaneously in an iterative process [45]. To initiate discourse, focus group discussions began with a provocative stimulus, a fictional case study describing typical situations that might arise in everyday interprofessional care [46, 47] (see Fig. 1). The use of such action-oriented exercises (pictures, provocative statements, etc.) has been shown to yield a deeper information than “normal” focus group discussions [47]. In the course of the discussion, the group’s shared background of experience and common patterns of cooperation and communication were reproduced and collective patterns of orientation and legitimacy in relation to the management of diversity in nursing were reconstructed. Group participants were able to adapt to central themes in a self-directed way.

In addition, an exmanent question section addressed relevant contexts, especially intra- and interprofessional cooperation in everyday practice. This was prepared in case the discussion came to a standstill or if the group does not deal with relevant issues on its own initiative.

The focus group discussions were audio recorded. Transcription of the data was carried out by an external contractor (professional transcription service). The transcripts were then pseudonymised and the audio files were deleted. In addition, a postscript was written after each discussion to provide contextual information. This included socio-demographic data (age, gender, qualifications, migration status) of the participants, characteristics of the group and the discussion, the seating arrangement and comments on non-verbal statements that cannot be captured by audio recordings. This allowed us to deepen the analysis by contextualising the course of the discussion, particularly with regard to the professional background of the participants and the relationships between the team members. The translation of the listed quotes for the purposes of this article was carried out by the author MS. For transcript analysis, ATLAS.ti (a computerised indexing system, GmbH, Berlin, Germany) was used.

In addition to five focus groups from the CareTrans project, four unpublished focus group transcripts from the TOP project (exploring the post-migration experiences of nurses in Germany) were used for this study. The decision to include old transcripts was based on the similarity of aims between the two projects. Including the transcripts from the earlier project provided a broader and more comprehensive dataset, allowing a deeper exploration of the nurse experiences and facilitating the identification of common themes and patterns across different time periods (for discussion stimulus ToP see Appendix).

### Qualitative content analysis

Depending on the degree of inductive reasoning, there are different approaches to qualitative content analysis [48, 49]. In conventional qualitative content analysis, coding categories are derived directly and inductively from the raw data, often used in grounded theory development. The second approach, which we used in this exploratory study, is directed content analysis, the purpose of which is to extend a conceptual framework or theory, in our case the conceptual framework of Schlossberg’s 4 S model [49]. Beginning with initial coding based on a pre-existing theory or relevant research, transcripts were analysed deductively using the four dimensions of transition theory according to Schlossberg’s Four S (4 S) framework as a priori items to construct the coding framework (Fig. 1). While a deductive approach to qualitative analysis begins with an organizing framework derived from existing literature, it is important to emphasize that this approach does not aim solely to validate predefined concepts. Rather, it provides a structured starting point for analysis that allows researchers to explore data with an awareness of established themes while remaining open to discovering new insights that may go beyond or even challenge existing knowledge. In the current study, a deductive framework was used that was informed by previous research and the specific research questions. This framework served as a guide for the initial coding as it allowed for a systematic engagement with the data based on what is already known about the phenomenon under study. In this way, the analysis was both structured and flexible, ensuring that the coding process was grounded in the existing literature but also open to the emergence of themes that went beyond the initial framework [50].

The authors then immersed themselves in the data, allowing additional themes to emerge organically [49]. Emergent themes were not included in the current study as they were beyond the purpose and research question. The decision to focus on themes that directly addressed the research objectives is consistent with the principle of analytical rigor, which emphasizes the importance of a coherent and focused qualitative analysis. This approach ensures that the findings are robust, credible, and directly relevant to the research question, which is central to producing high-quality qualitative research [51].

We followed the main stages of qualitative content analysis starting with decontextualization, where the author (MS) broke down the transcripts into smaller parts and assigned codes to key phrases or concepts that seemed significant and relevant to the research question based on six focus group transcripts, after which data saturation was reached [52]. This was followed by recontextualization, in which the author LP reviewed the initial codes to ensure that they accurately reflected the data and ensured that the context of the data was preserved. In the categorization phase, the authors MS and LP grouped similar codes into themes to identify patterns within the data, and in the final compilation phase, the relationships between themes were explored to draw conclusions and insights. Overall, the codes were “revised and modified iteratively to reflect the data” [37]. Throughout the various stages, all authors regularly reviewed the coding and interpretation of the data, with all authors resolving any differences in interpretation through discussion and negotiation, followed by a consensus process among all authors [53].

### Quality, rigour, and reflexivity

Reflexivity about the relationship between researchers and participants is crucial to maintaining the quality of the research [54]. In this study, the researchers take an outsider’s perspective, lacking direct experience within German nursing teams. On the one hand, this approach has potential advantages, such as promoting a more objective coding and analysis process free from personal bias. On the other hand, being an outsider can pose challenges in terms of understanding the nuances of nursing teams and establishing rapport with participants. As an interdisciplinary research team with diverse professional, national, and cultural backgrounds, as well as varying levels of experience with qualitative studies, we were reflective about the implications this might have had on the depth and breadth of the study, allowing for a more comprehensive understanding of the research topic and potentially yielding more robust and nuanced findings.

Credibility is the qualitative equivalent of internal validity and was established through various means: Triangulation was achieved by describing the analytical process in detail and analysing the data by more than one person (researcher triangulation), as well as by including more than one hospital site in Germany as well as different participant backgrounds (German, non-German, and vocationally or academically trained) (data triangulation) to ensure comprehensiveness and more reflexive data analysis [55]. In addition, validity was ensured through constructive alignment between the research questions, the epistemology underpinning the research, and the methods used [56].

### Ethics

The study received a positive ethical vote from the Ethics Committee of Charité - Universitätsmedizin Berlin (CareTrans EA1/260/21, Top EA1/062/18). A comprehensive data protection concept was developed and reviewed by the responsible staff unit. For data collection under the conditions of the corona pandemic, a hygiene concept was prepared and reviewed by the responsible hygiene officer. Written informed consent was obtained from all participants.

## Results

### Participants

Nine focus group discussions were held. A total of 40 nurses from different educational and migration backgrounds participated. Participants included German and migrant nurses from different countries of origin working in six hospitals in two German federal states. Four of the hospitals are denominational (associated with a particular religious denomination) and two are municipal (under the control of a local government).

The nine group discussions were conducted by PB and LP. Due to the numerous languages of origin of the participants, the decision was made to conduct the group discussions in German – the only language that all participants could speak, albeit at different levels.

The focus group discussions took place in the premises of the respective institutions and lasted between 35 and 66 min. The demographic information of the study participants and focus group discussions is summarized in Table 1.

**Table 1:** Demographic information of the study participants

Focus group	Duration	Participants			
		Number	Gender: Female/ Male	Location of nursing training: German trained/ Foreign trained	Education background: Vocational training/ Academic education
FG A	0:38	4	3/1	3/1	4/0
FG B	1:06	4	4/0	4/0	3/1
FG C	0:50	4	4/0	3/1	4/0
FG D	0:35	4	2/2	3/1	3/1
FG E	0:40	7	6/1	5/2	5/2
FG F	0:41	4	2/2	0/4	4/0
FG G	0:41	4	2/2	4/0	2/2
FG H	0:40	3	3/0	2/1	3/0
FG I	0:57	6	6/0	1/5	5/1
**Total**	**6:08**	**40**	**32/8**	**25/15**	**33/7**

### Coding framework

The final coding framework was composed of the four dimensions of transition theory according to Schlossberg’s Four S (4 S) framework. The themes were self, situation, strategies, and support (Table 2).

**Table 2:** Coding framework

Theme	Description according to Schlossberg	Extension to team perspective
**S**elf	This theme is related to professional identity, roles, values, and self-perception.	Team perspective on collective professional identity, team experiences and expectations, and shared team values, roles and perceptions.
**S**ituation	This theme is related to the change event and the external environment, such as labour market conditions or societal changes.	Team perspective on the dynamic educational, professional and cultural landscape, the academicization of nursing, the shortage of qualified nursing staff.
**S**upport	This theme is related to the social and interpersonal resources, including the networks, relationships and support from others.	Team perspective on the dynamics within nursing teams, including attitudes towards changing roles.
**S**trategies	This theme is related to actions and coping mechanisms, behavioural adjustments and steps to address challenges.	Team perspective on the coping mechanisms and institutional and peer strategies for adaptation.

### Theme 1: Self

**Fig. 2:**
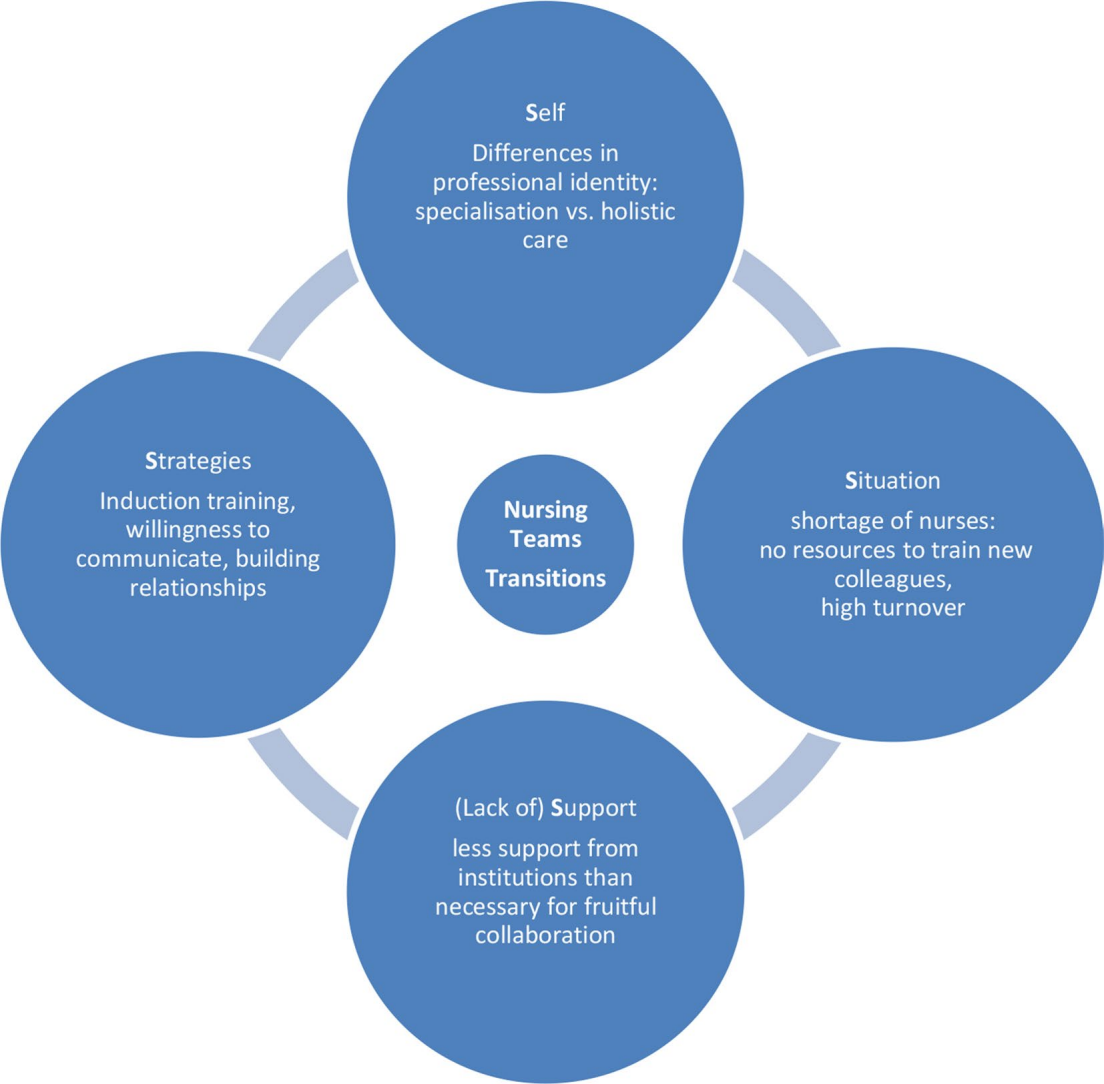
Summary of the results (adaptation of Schlossberg’s Four S (4 S) framework to a team perspective)

In the original framework, this theme explores personal characteristics such as prior experiences, expectations, roles and aligned values. For the purpose of this study, we also considered participants’ professional identity, education/training and migrant background. In terms of the team perspective, this includes team experiences and expectations as well as shared values and a collective identity as a nursing team (Fig. 2).

The analysis showed that the expectations of the professional role of an academized nurse are closely linked to previous experience in Germany or abroad. Participants of German origin have more realistic expectations of their professional development and identity:

*“Well*,* I’m a university graduate myself*,* so I can say that … I’m doing exactly the same job as everyone else. But I think that’s fair. Well*,* I knew from the beginning that this is the way it is here in Germany. And I don’t see myself any differently than my colleague.” (FG B*,* female participant*,* birth and academic education in Germany)*.

The participant considers it fair that she does the same work as her colleagues, despite her formal higher education. On the one hand, she justifies this with the established structures in German nursing and her knowledge of them. On the other hand, she justifies it with an egalitarian orientation that puts her on the same level as her colleagues. This attitude can also be found among other nursing staff who have received academic training in Germany. Our data includes only one international nurse who also shows this attitude; this is because he has already worked in an intensive care unit in his country of origin:

*“So washing is actually okay for me*,* because I [was] in the intensive care unit*,* because we also do washing like that. But for example*,* if you actually work in the normal wards*,* so as I said*,* one relative stays for the whole night next to the patient’s room.” (FG D*,* male participant*,* birth and academic education abroad).*

Altogether, this attitude may also be due to the fact that both the German trained and the foreign trained nurse work in an intensive care unit where the tasks and responsibilities of the nursing staff are more homogeneous – even in different countries – than on other wards, as the observation protocols underline.

In contrast, most migrant participants expressed a general sense of disappointment and discomfort with the change in their professional identity, which they attributed to the fact that they had previously been recognised as “doctors” and had a more respected professional image in their home countries than the perception of nurses in Germany. The context of origin acts as a comparative horizon:

*“I would have had a good future as a nurse in my home country if I had worked there. … I’m seen as a doctor there because that’s how I work*,* I work as a doctor.” (FG F*,* female participant*,* foreign-born and academic education abroad)*.

A clear divide emerged within the study cohort: on the one hand, the foreign nurses who felt that their academic qualifications were devalued in German nursing, and on the other hand, the German nurses who perceived a sense of superiority on the part of their foreign colleagues. At the centre of these differences is the professional profile of nursing, usually in relation to the medical profession. This is illustrated in particular by the following quote from a team that recently had a conflictual experience with an academically trained foreign nurse who is now working on another ward:

*“That is the problem. They think that with their academic degree they are better than us with our education here in Germany. And that’s not the case. I think that’s a very fatal mistake with the expectations they bring here. They are not doctors! (FG E*,* female participant*,* birth and academic education in Germany)*

At the heart of this distinction is professional identity; while in Germany, despite (or because of) the major changes, the ideal image of traditional holistic nursing dominates, in most other countries nursing is characterised by specialisation and hierarchies. This is associated with correspondingly different responsibilities in everyday work:

*“Things are different here (in my homecountry). For example*,* the nurses don’t wash the patients*,* it’s the nursing assistants who do that here. We do a lot of bandaging*,* for example. We have to organise a lot of things and lead the team.” (FG I*,* female participant*,* foreign-born and academic education abroad)*.

This migrant nurse reports that, in her country of origin, professional nurses take on responsible and medical tasks, while care assistants provide basic care. In Germany, according to the holistic care paradigm, professionals are responsible for basic care. In public discourse, however, basic care is seen as a simple or even “dirty” activity. In the focus groups, German nurses repeatedly referred to this, especially when anticipating the views of their international colleagues:

*“He probably worked in the operating theatre there (in the home country). Now he has to wash people here*,* he must think it is dirty work. He doesn’t want to do it. (FG G*,* female participant*,* birth and academic education in Germany)*

Overall, it can be seen that professional identity and expectations within nursing teams are significantly influenced by cultural and educational backgrounds, highlighting the importance of understanding and addressing different professional profiles and expectations in order to promote better integration and cohesion within nursing teams.

### Theme 2: Situation

In the original framework this theme explores factors related to the change event, such as the triggers, duration and impact of the change (improved or worsened status). In our sample, it was extended to the team’s experience of change, mainly driven by the dynamic educational, professional and cultural landscape of the health professions in general and the academicization of the nursing profession in particular (Fig. 2). Nursing teams need to work together to manage the profound changes in their daily work. The shortage of qualified nursing staff is acting as a catalyst, increasing the pressure on all concerned to adapt.

There was a consensus among the participants (both German and foreign) about the dynamic labour market situation in Germany, mainly due to increasing job dissatisfaction and the resulting high turnover of nurses. German nurses felt that the main difficulty was that teams were constantly having to train new colleagues, but did not have the human resources to do so:

*“The work situation is getting more and more difficult. New nurses are coming in and they need to be trained. Experienced nurses who used to be there are not always there … I think the mix has come about because a lot of colleagues have left. As a result*,* there have been a lot of new recruits recently*,* and there have always been colleagues with an immigrant background.” (FG G*,* female participant*,* birth and apprenticeship training in Germany)*.

As a result, there was a lack of preparedness on the part of the new foreign nurses, which in turn exacerbated the high turnout and frequent changes of employer:

*“No*,* we recently had a colleague on our ward who came from Brazil. I think a lot of people came to Germany from Brazil at that time. She started working in a university hospital and then somehow she was transferred to us. And then*,* yes*,* she couldn’t cope either. Because she had also studied nursing in Brazil and she didn’t understand what she had to do here and what was expected of her … And in the end she didn’t make it*,* she was dismissed.” (FG I*,* female participant*,* foreign birth*,* apprenticeship training in Germany)*.

In terms of the impact of change, most of the participants in the study experienced a deterioration in status and devaluation rather than an improvement, adding to the general sense of disappointment. They feel a double devaluation, both of their education and their professional experience. Some participants even felt that they had lost all their experience and were starting from scratch:

*“I have learnt something else. And I’ve been doing it for four years*,* so I’ve studied for four years and now I have experience. But I still don’t feel like I have four years of experience*,* because it’s just different. So it was like I learned something different.” (FG I*,* female participant*,* foreign birth and academic education abroad).*

The dichotomy in educational backgrounds within the nursing workforce was evident within the study cohort and contributed to different perspectives on the competence and practice readiness of colleagues. Participants witness the increasing trend towards academic education among nurses in the German context:

*“It’s understandable when you know that everyone on the ward*,* every second nurse*,* goes to university. I already know 4 colleagues who have started their nursing studies at university.” (FG G*,* female participant*,* birth and academic education in Germany)*.

The increasing academisation in Germany may have an impact on the perception of professional competence. Other participants expressed concern about a university degree without sufficient practical experience and the need to retrain such colleagues. It is rather the German nursing staff with non-academic training who criticise the lack of practical experience of their foreign academically trained colleagues, as the following example on shortcomings in hygiene practices shows:

*“We also have students from Albania who have completed their university studies*,* of course they have completed many semesters*,* but without any practical experience. And*,* as you said*,* in terms of hygiene*,* unfortunately*,* a complete disaster. And unfortunately*,* you have to train them all over again.” (FG G*,* male participant*,* birth and apprenticeship training in Germany)*.

Overall, it can be seen that the already high workload of the nursing teams is further increased by the need for additional training of foreign colleagues. Due to the shortage of qualified staff, the nursing teams do not have sufficient resources to train the new colleagues, who are therefore unable to develop their nursing potential. The result is a high turnover of foreign nurses, which ultimately leads to a downward spiral.

### Theme 3: (lack of) support

In the original framework this theme explores the different types of support (personal or institutional) for individuals undergoing transitions. In our study, we extended it for nurses experiencing change in the current professional landscape to include the dynamics within teams, including attitudes towards changing roles (Fig. 2).

While politicians and hospitals see international nurses as a support to the nursing teams, the organizations offer relatively little support in the onboarding process. The following example shows that neither the migrant nurses nor the teams are involved in deciding who should work together:

*Dw: aren’t they asked beforehand where they want to go?*

*Bw: No*,* that is decided.*

*(FG E, female participants, birth and apprenticeship training in Germany)*

This team works in a specialized area where many patients are in need of care, so basic care is a large part of their daily work. As mentioned above, this activity often leads to misunderstandings within the team. This team clearly states that they lack support from their organization:

*Cw: Yes. You just often feel left alone …*.

*Aw: … so abandoned …*.

*Cw: They come here … and then as a specialist you have to …*.

*Aw: … do it!*.

*Cw: … … train them here*,* in addition to our work. That’s already … Someone else would have to be on hand. (FG E*,* Aw and Cw both female participants*,* birth and apprenticeship training in Germany)*

Participants emphasised the considerable need for institutional support for migrant nurses by their employers as early as possible, i.e. in their home country before they are even recruited to have realistic expectations about the situation in Germany:

*“I think it would have been important to say at the time of recruitment: ‘We know it’s a different job in your country. If we bring you to Germany*,* you can expect this and that. … It’s also simply a question of what they are told*,* or in other words*,* what is initially conveyed to them when they come here to Germany. They are told about the wonderful life here and*,* well [sighs]… it’s a challenge. … when you’re starting somewhere new*,* so I could imagine that it’s important to get an understanding of the profession first*,* but maybe the colleagues in the team also understand where this person is coming from.” (FG F*,* female participant*,* foreign birth*,* apprenticeship training in Germany)*.

The participant herself has a migrant background and has many contacts with care professionals who work in other countries. She therefore adopts a mediating perspective here and emphasises that both the new and the old team members need to be better informed at an early stage, which could make cooperation more difficult. She therefore talks about improving expectation management for both sides.

In other teams, too, it is problematised that a lack of knowledge on the part of long-established team members can be a source of potential conflict:

*“So it sounds to me like there has been a conflict for some time…. Does the team know where they (international nurses) come from*,* what kind of training they have?” (FG B*,* female participant*,* birth and apprenticeship training in Germany)*.

However, a general lack of understanding of the professional backgrounds and previous roles of migrant nurses led to conflict and difficult team dynamics.

Overall, it can be said that the nursing teams receive less support from the institutions when integrating new professionals and in their day-to-day work than would be necessary for fruitful collaboration. The nursing teams must therefore find their own ways of integrating the different qualifications, training content and job profiles of those involved into the processes.

### Theme 4: Strategies

In the original framework this theme explores the strategies and coping responses developed by individuals in transition situations. In our study it was extended to the nursing teams, including the tactics used to manage change successfully (Fig. 2). It should be emphasised that all participants assume that the migrant nurses will adapt to the existing structures and processes. Rather than discussing how the migrant nurses can contribute the skills they have already acquired, the participants focus on the fact that their non-existent skills threaten the status quo. This problem-centred perspective means that ‘supporting’ refers to helping to adapt.

Two teams use an onboarding approach where each new nurse is properly trained in their new role. Interestingly, these are the two teams that are relatively well staffed. The following example also shows the positive impact that induction training by established migrant colleagues can have:

“*Bm: I think that also comes from induction training.*

*All: Yes.*

*Bm: Everyone starts with someone who is experienced here*,* who knows the processes … And that’s not a question of (.) academic or non-academic qualifications.*

*Dw: But I have to say that I was trained by [Sonia]*,* (.) she is Spanish*,* and with her I realized very clearly how much she knows and how many details she can say in the background to explain things … I was very*,* uh*,* impressed by @(.)@ how much she actually knew.*

*(FG A*,* Bm: male participant*,* birth and apprenticeship training in Germany; Dw: female participant*,* born abroad and apprenticeship training in Germany)*

Dw is impressed by her Spanish colleague’s academic expertise, and the other participants agree with her as the discussion progresses. This is notable because this team is an exception in this sample. This also applies to the willingness to accommodate international nursing staff, even though the established nursing staff are calling the shots:

*“Look*,* I’ve been working here for 15 years; I know exactly what to do. And now you come and tell me that in your country you can take blood samples and put in cannulas or whatever all the time. You can do that here too*,* but let’s start with basic care before we get to the medical side.” (FG B*,* female participant*,* birth and apprenticeship training in Germany)*.

Nevertheless, all groups see communication as a central aspect of good collaboration strategies. This, of course, relates to language skills, which is a recurring problem for the teams, but is mostly expected to be solved by the individual nurses. In the team context, it is about the willingness to communicate with each other, even if the German language skills are poor. However, this does not always lead to satisfaction, as the following example shows. An international nurse had been sent to the team by the hospital management, but the collaboration did not work at all. The established members of the team tried to communicate, but withdrew in disappointment when this did not bring about any improvement:

*“But I think at that point you don’t really have the motivation anymore. Because you see… well*,* of course*,* there’s no question*,* if they really come and show interest*,* of course*,* no question*,* but I find that if they really show no interest and then you have to go and say something [grimaces]… that’s how I see it. If I see*,* okay*,* no interest*,* then I’ll let it go*,* I’ve got to be honest.” (FG E*,* female participant*,* birth and apprenticeship training in Germany)*.

The management also tried to speak to the nurse in question:

*“Well*,* I really tried*,* so as I said*,* my patience is long*,* it’s really very long*,* but at some point it really … Then there was another conversation with our practice coordinator and this integration officer and*,* um*,* now here [our ward manager] and our [international] nurse.” (FG E*,* female participant*,* birth and apprenticeship training in Germany)*.

In the end, the nurse was transferred to another ward in the same hospital. This example shows that sometimes it just doesn’t work out - even when the people involved make an honest effort. Unfortunately, we don’t know the international nurse’s perspective. However, it is reasonable to assume that such disappointing experiences could have been avoided if there had been more support from the hospital beforehand - at best in the form of a say in the choice of location.

Such stories get around and cause serious damage, undermining confidence in the process:

*“But in the [other hospital] it was also the case that the 15*,* I was at a congress recently*,* it was also about the fact that they brought 15 nurses*,* I don’t even know where they got them from*,* and with language school and everything*,* and all 15 went back after language school*,* after training.”(FG G*,* male participant*,* birth and academic education in Germany)*.

This is central to the fact that in our sample, the expected length of stay at a workplace plays a crucial role in shaping the strategies used to build professional relationships; participants who regularly rotate through different departments are less motivated to engage in sustained relationships with colleagues:

*“Moderator: If I know I have to work with them for the next ten years*,* then I try to establish a working relationship*,* and then I am also friendlier.*

*Aw: That’s true. We used to have a solid*,* fixed team. The senior doctors used to switch roles every six months*,* going back to the operating room*,* but that was different.” (FG B*,* female participant*,* birth and apprenticeship training in Germany)*.

German participants, in particular, emphasised the need for a comprehensive strategy to address the challenges of foreign-trained colleagues, and how the lack of such a strategy can lead to disillusionment and the decision to lose colleagues who return home.

*“The same thing happened to those who came from Mexico and Spain. They all went back and said: ‘Hey*,* please! I’m not doing this job for this money!’ [Agreement from the others].” (FG E*,* female participant*,* birth and academic education in Germany)*.

Overall, it can be seen that the nursing teams apply the key strategies of on-boarding and communication - but the situation in nursing (shortage of skilled workers and high fluctuation, especially of international nursing staff) as well as the lack of support from institutions usually stand in the way of successful implementation. Although the teams are motivated, they simply lack the supportive framework conditions to implement the strategies.

## Discussion

The global migration of health professionals in general and nurses in particular, is a growing phenomenon that is constantly evolving with the ongoing changes in global societies and health systems. The present study provides insights into the experiences and challenges faced by nursing teams in the current dynamic scene of migration, academicization and professional socialisation in Germany. Disparities in the experiences of nursing teams in Germany arise from elements such as professional role confusion, significant turnover and differing views on the academicization of nursing education. Taken together, these factors foster feelings of professional devaluation and disappointment within the nursing community. In the next section, we will analyse our findings within the sub-domains of self, situation, support and strategy, as adapted from the Schlossberg 4 S framework, and contextualise them in relation to the existing literature. Furthermore, we will formulate recommendations aimed at improving the experiences of nursing teams based on the challenges identified in our sample.

The exploration of the self-dimension revealed that the impact of participants’ migrant or German background in shaping their experiences was greater than that of their educational background. While nursing team members of German origin consistently expressed realistic expectations of their professional identity, thus acknowledging established norms in Germany, migrant team members expressed dissatisfaction and a disparity between their valued professional image in their home countries and the perception of nurses in Germany, leading to a sense of professional devaluation [16, 57]. This is consistent with findings from other studies indicating that foreign-trained nurses find themselves in roles that devalue their previous training and experience, to the point that they give up their dream of becoming a registered nurse [58]. Beyond personal dissatisfaction, the impact extends to health system resources, as the underutilization of the education and skills of foreign-trained nurses represents an inefficient allocation of valuable resources and adds to the already overburdened working conditions of nursing teams [59]. The literature also shows that, regardless of cultural background, common stressors such as lack of recognition, and emotional challenges associated with caring for patients are universally perceived as distressing. However, in a cross-cultural team work, the challenges are compounded by different interpretations of behaviors, different approaches to care, and communication barriers, which together make it difficult for a culturally diverse nursing teams to work together effectively [60]. A concrete example from the literature is the obligation to adhere to strict schedules and meticulous documentation, which is described by migrant nursing team members as “German punctuality” and the “strict German way of working” as a burden. In their view, flexibility in communication rather than rigid adherence to paperwork is necessary for effective nursing care. Conversely, German nurse team members feel pressured by what they perceive as the “lax mentality” of migrant nurses in terms of time management, which in their view increases the time pressure in the workplace [60].

The different perspectives on professional identity highlight the nuanced nature of nursing roles within different contexts, and the global variation in definitions and practices associated with the term ‘nurse’, as acknowledged in the literature [2, 61]. Depending on the country of origin, migrant nurses will have different degrees of autonomy in the assumption of patient responsibility; this discrepancy will lead to a shift in professional identity, which can have a major impact on team dynamics and assimilation in the workplace [62]. A distinctive feature of the German nursing landscape is the sharing of responsibility for basic care activities, such as bathing and feeding patients, between both vocational and academic nurses [63]. This contrasts with the prevailing practice in many other countries, where such tasks are typically delegated to nursing assistants or performed by patients’ relatives [64].

The examination of the situation theme in our study sheds light on the complex nature of the dynamic health care landscape within the nursing profession in Germany, where nursing is undergoing a process of change from a formerly “female” profession to one whose members are increasingly diverse in terms of professional socialization, qualification profiles and cultural backgrounds. The shortage of qualified staff is acting as a catalyst, accelerating this process and leading to challenging labor market dynamics, mainly driven by increasing job dissatisfaction, high turnover of nurses and frequent changes of employer [65–67]. In addition, the dichotomy of educational backgrounds within the nursing workforce emerged as a significant issue, influencing perceptions of professional competence, and requiring a careful balance between academic education and practical experience [67, 68]. This is consistent with existing research documenting considerable variation in the quality, scope and duration of nurse education and preparation systems [59].

Analysis of the theme of support provides important insights into the challenges nursing teams face in a changing professional landscape. Participants generally emphasize the importance of comprehensive professional support for nurses, regardless of their educational or migrant background [69]. German participants, in particular, emphasize the need for early institutional support for migrant nurses and advocate realistic expectations prior to recruitment to address differences in nursing practice between countries [69]. This perspective adds a valuable dimension to the existing literature. While current research often emphasizes the preparation of nursing teams in destination countries to receive foreign-trained nurses, our findings contribute by highlighting the importance of training foreign-trained nurses as well [70]. This dual perspective underscores the importance of ensuring clear expectations and readiness on both sides of the integration process and enriches the understanding of effective support mechanisms in the context of international nurse migration.

Personal support from colleagues is seen as essential, as a lack of understanding of migrant nurses’ experiences and roles leads to conflict and challenging team dynamics [71]. This finding is consistent with the literature, where foreign-trained nurses often emphasize that the most important form of support comes from their peers, particularly other international nurses [72].

Analysis of the strategy theme revealed intra-organizational and sociocultural strategies for successfully managing change, consistent with similar findings in the extant literature [73]. In contrast to the wide range of intra-organizational strategies in the literature (e.g., policies, diversity initiatives, treatment of employees), our sample was limited to collegial social and professional interactions and peer support, which emerged as a prominently mentioned strategy [74]. In addition, the expected length of stay at a job emerged as a key factor shaping the professional relationship-building strategies of our sample. Participants who frequently rotated through different departments expressed less motivation to engage in sustained relationships with colleagues, citing a lack of connection and the transient nature of their positions.

In our sample, learning German emerged as an important socio-cultural strategy. Notably, in contrast to the literature, there was no mention of cultural training, learning and social support, or personal skill development [73]. The fact that internationally trained nurses face the additional challenge of learning a new language while adapting to a new healthcare system appears to be a significant stressor to them and their teams [16]. These aspects mean that international nurses in Germany are more likely to work overtime, are disadvantaged in the distribution of work tasks in everyday working life - regardless of their level of qualification [21].

Building on these findings, it is recommended that policy and practice focus on enhancing the integration and support of nursing teams within Germany’s evolving healthcare landscape. At the national policy level, this could include the development of targeted support programs that address the unique challenges faced by migrant nurses, such as implementing inclusive professional development initiatives and fostering a deeper understanding of diverse professional identities. Specific interventions within healthcare organizations should prioritize the expansion of intra-organizational strategies, including recognizing the impact of expected length of stay on professional relationships and emphasizing language and cultural training. In addition, promoting a culture of peer support and inclusivity within care teams is critical to creating a more supportive and efficient healthcare environment. These policy changes and organizational strategies may contribute to the successful integration and satisfaction of nursing teams, thereby optimizing the use of skills and resources within the healthcare system. Future research should examine the effectiveness of these interventions and further explore the role of national and organizational policies in shaping the experiences of nursing teams.

### Limitations of the study

Language was the most significant limitation of this study. Although the literature recommends conducting focus group discussions in the native language of the participants, this was not feasible in the current research [45, 56]. The participants within each focus group had different native languages, making it impractical to conduct discussions in their respective native languages. Consequently, the focus groups had to be conducted in German. This linguistic diversity posed a limitation to the study, as German participants were free to express themselves, while international participants may have faced challenges due to varying levels of German proficiency, potentially affecting the depth and accuracy of their contributions. To mitigate this problem, the researchers documented the study participants’ nonverbal communication in the postscript, including gestures and facial expressions, which helped to capture additional nuances and ensure a more complete understanding of their perspectives in addition to their verbal comments. Females are overrepresented in our sample (the target population of nurses in Germany is 75% female compared to 25% male), which may have implications for the generalizability of our findings. While we acknowledge the gender imbalance in our sample as a limitation, we recommend that future research focus on recruiting a more gender-balanced sample to better represent the nursing population in Germany. In addition, exploring gender-specific experiences and perspectives within the nursing teams could provide valuable insights and increase the generalizability of findings to both male and female nurses. Future studies could also consider examining other demographic factors, such as age, to address potential limitations and broaden the understanding of different experiences within nurse teams. In addition, future research could explore how nurse teams’ transition through the different stages of team building, especially since there is a high turnover rate.

## Conclusions

This research provides an overview of the experiences and challenges faced by nursing teams in Germany as a result of global migration trends and the process of academicization. The study extended the Schlossberg 4 S framework from individual to collective experiences of nursing teams, providing a broader perspective on the challenges and adaptations encountered. Across all domains of the framework, the experiences of nursing teams can be characterized as heterogeneous.

The coexistence of nurses with diverse educational, professional, and cultural backgrounds in healthcare can be a management challenge, but it is also considered a strategic asset. This diversity is not only enriching for healthcare teams, but also beneficial as patients and care recipients become more diverse. Matching the diversity of healthcare teams with the changing demographics of the population they serve is seen as a valuable aspect that has the potential to improve the quality of healthcare services provided.

## Electronic supplementary material

Below is the link to the electronic supplementary material.

Supplementary Material 1

## Data Availability

The datasets used and/or analyzed during the current study are available from the corresponding author on reasonable request.
