# Supplementary material for: Navigating transitions: a qualitative study of nursing teams’ experiences of educational and cultural transitions in Germany
Source: BMC Nurs. 2024 Oct 8;23:725. doi: 10.1186/s12912-024-02383-0 (PMC11463137; doi:10.1186/s12912-024-02383-0)
Supplement: Supplementary file 1 — Supplementary Material 1 [file 12912_2024_2383_MOESM1_ESM.docx]

**Navigating Transitions: A qualitative study of nursing teams' experiences of educational and cultural transitions in Germany**

**Appendix**

**Discussion stimulus ToP: The multicultural ward**

On the occasion of the planned new employment of a Mexican staff member in the multicultural ward, a staff member, Mr. Paulsen, expressed concerns. Mr. Paulsen drew the management's attention to possible problems in the team if "the migrant issue continues to be handled so naively". He said that a certain degree of cultural mixing should not be exceeded in the team: "It is exciting with the different nationalities, but I have the feeling that at some point it will tip over. We can't take another one, because of the group dynamics.
